# Supplementary material for: Prefrontal cortex molecular clock modulates development of depression-like phenotype and rapid antidepressant response in mice
Source: Nat Commun. 2024 Aug 23;15:7257. doi: 10.1038/s41467-024-51716-9 (PMC11344080; doi:10.1038/s41467-024-51716-9)
Supplement: Supplementary file 1 — Supplementary Information [file 41467_2024_51716_MOESM1_ESM.pdf]

Supplementary materials for

Prefrontal cortex molecular clock modulates development of depression-like phenotype and rapid antidepressant response in mice

Sarrazin & Gardner et al.

Supplementary Figures 1-7 and Legends:

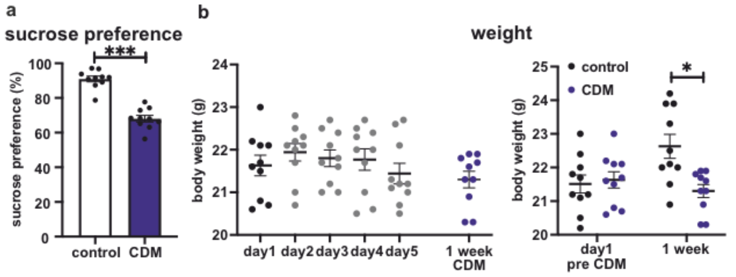

c

Clock genes	*Per1*	*Per2*	*Cry1*	*Cry2*	*Bmal1*	*Clock*	*Npas2*	*Rev-erbα*	*Rorα*	*Rorβ*	*Rorγ*
Rhythmicity control	*P*=0.0003	*P*<0.0001	*P*<0.0001	*P*<0.0001	*P*<0.0001	*P*=0.1817	*P*<0.0001	*P*<0.0001	*P*=0.0001	*P*<0.0001	*P*<0.0001
Rhythmicity CDM	*P*=0.0010	*P*<0.0001	*P*<0.0001	*P*<0.0001	*P*<0.0001	*P*=0.8098	*P*=0.0021	*P*=0.0007	*P*=0.0408	*P*<0.0001	*P*<0.0001
cntr vs CDM	*P*=0.0731	*P*<0.0001	*P*=0.2122	*P*<0.0001	*P*<0.0001	*P*=0.9771	*P*=0.6904	*P*<0.0001	*P*<0.0001	*P*<0.0001	*P*=0.0827
Amplitude cntr vs CDM	*P*=0.6973	*P*=0.0066	*P*=0.0358	*P*=0.0071	*P*=0.6374	-	*P*=0.5432	*P*=0.2048	*P*=0.0421	*P*=0.2551	*P*=0.2538
Acrophase cntr vs CDM	*P*=0.346	*P*=0.0082	*P*=0.0402	*P*<0.0001	*P*<0.0001	-	*P*=0.0650	*P*=0.0074	*P*=0.0005	*P*=0.1109	*P*=0.0681

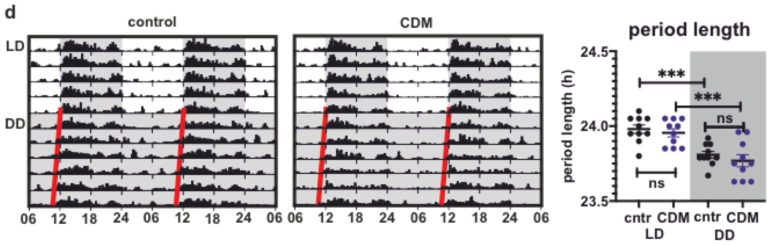

e

Clock genes	*Per1*	*Per2*	*Cry1*	*Cry2*	*Bmal1*	*Rev-erbα*	*Rorα*
Rhythmicity control	*P*<0.0001	*P*<0.0001	*P*=0.0002	*P*=0.8886	*P*<0.0001	*P*<0.0001	*P*=0.0267
Rhythmicity CDM	*P*<0.0001	*P*<0.0001	*P*=0.0046	*P*=0.4863	*P*<0.0001	*P*=0.0068	*P*=0.0011
cntr vs CDM	*P*=0.8160	*P*=0.6856	*P*=0.1748	*P*=0.2022	*P*=0.1367	*P*=0.0597	*P*=0.1210

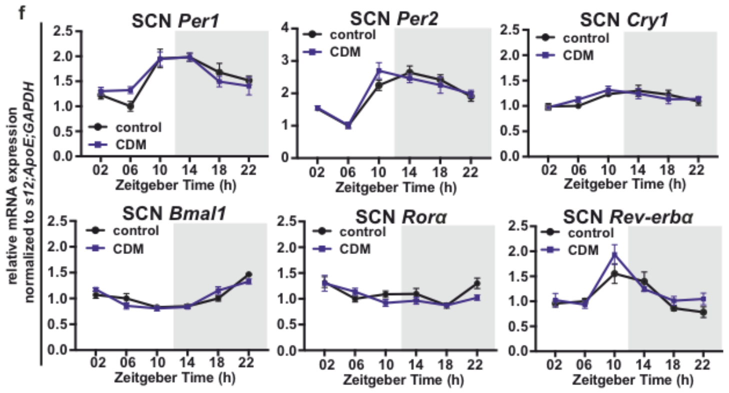

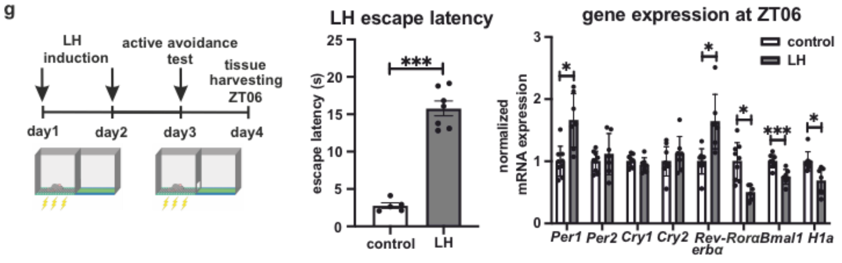

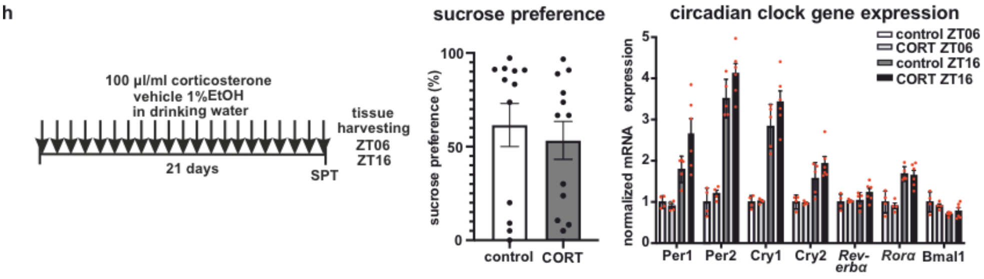

Supplementary Figure 1

Supplementary Fig. 1

a Nosepoke sucrose preference of control and CDM mice one week after CDM induction phase (n=10 mice per group, two-tailed Student's t-test: ****P*<0.0001).

b Mice weight during the induction phase and test phase 1 week later (left) and the weight of naïve and CDM mice before the CDM and 1 week after the CDM protocol (right) (n=10 mice per group; repeated measures ANOVA with Bonferroni post-hoc test: **P*<0.05).

c Comparison of statistical significance: rhythmicity, CDM effect (two-way ANOVA, control vs CDM), amplitude and acrophase (control vs CDM, determined via nonlinear regression sine wave fit and cosinor analyses) for each analyzed clock gene in the mPFC (see also Fig. 1b)

d Representative double plotted actograms showing the locomotor activity of control and CDM mice in IntelliCage (left) and circadian period length (right) at 12:12h LD and complete darkness (DD) (n=10 mice per group, two-way ANOVA with Bonferroni post-hoc test: ****P*<0.001).

e Comparison of statistical significance for rhythmicity (determined via nonlinear regression sine wave fit and cosinor analyses) and total expression (CDM effect, two-way ANOVA) for each analyzed clock gene in SCN**.**

f Relative mRNA expression of clock genes in SCN samples harvested from naïve (control) and CDM mice every 4h under 12:12h LD conditions. (n=5 mice per group, two-way ANOVA).

g Experimental design of the learned helplessness model: procedure included two days of induction (inescapable footshocks) followed by a testing day (active avoidance) in a 2-chamber shuttle box and killing around ZT06 on day4 (left); Escape latency time of control (n=5) (without day1+2 LH induction) and LH mice (n=7) during the test day (middle) (two-tailed Student's t-test: ****P*<0.0001); relative mRNA expression of clock genes in mPFC at ZT06 of control (n=9: 4 LH controls and 5 naïve mice) and LH mice (n=7) (two-tailed Student's t-test: **P*<0.05, ****P*<0.0001). Data are presented as mean ±SEM and the individual data points are depicted.

h Experimental design of the chronic CORT model: mice were exposed to 100 μl/ml CORT in the drinking water for 21 days, on the last day classical SPT was conducted with 1% saccharine (left); sucrose preference of control and CORT treated mice (middle) (two-tailed Student's t-test); relative mRNA expression of clock genes in mPFC at ZT06 and ZT16 of control (n=4 & 5) and CORT mice (n=6) (two-way ANOVA with Tukey post-hoc test: P-values for ZT16 control vs CORT are indicated above the bars).

Data are presented as mean ±SEM and the individual data points are depicted.

a

Clock genes		*Per1*	*Per2*	*Cry1*	*Cry2*	*Bmal1*	*Rev-erbα*	*Rorα*	*Rorβ*
Rhythmicity KET		*P*<0.0001	*P*<0.0001	*P*<0.0001	*P*<0.0001	*P*<0.0001	*P*<0.0001	*P*=0.0005	*P*<0.0001
CDM vs KET		*P*<0.0001	*P*<0.0001	*P*<0.0001	*P*<0.0001	*P*=0.0041	*P*=0.0013	*P*<0.0001	*P*=0.9259
Amplitude	control	0.43±0.07	1.25±0.11	0.71±0.04	0.34±0.03	0.68±0.05	0.32±0.04	0.37±0.04	0.71±0.04
	CDM	0.35±0.06	1.90±0.11	0.52±0.06	0.48±0.04	0.73±0.05	0.40±0.05	0.20±0.05	0.52±0.06
	KET	0.33±0.05	0.32±0.07	0.42±0.07	0.54±0.06	0.81±0.10	0.25±0.05	0.38±0.10	0.42±0.07
	CDM vs KET	*P*=0.1454	*P*<0.0001	*P*=0.2920	*P*=0.0453	*P*=0.8264	*P*=0.8849	*P*=0.0844	*P*=0.8100
	control vs KET	*P*=0.3818	*P*=0.0049	*P*=0.0015	*P*=0.0005	*P*=0.5817	*P*=0.4015	*P*=0.4358	*P*=0.7455
Acrophase (ZT)	control	9.39±0.63	15.8±0.34	16.1±0.25	14.4±0.31	24.2±0.29	11.2±0.51	15.1±0.37	18.8±0.12
	CDM	10.1±0.46	16.9±0.23	16.8±0.51	16.8±0.51	20.7±0.24	9.23±0.48	11.6±0.90	18.5±0.06
	KET	7.25±0.65	19.9±0.92	23.2±0.54	21.9±0.42	22.9±0.44	7.73±0.88	14.9±0.99	16.9±0.83
	CDM vs KET	*P*=0.0071	*P*=0.0104	*P*<0.0001	*P*<0.0001	*P*=0.0051	*P*=0.8351	*P*=0.0981	*P*=0.3297
	control vs KET	*P*=0.1103	*P*=0.0007	*P*<0.0001	*P*<0.0001	*P*=0.1029	*P*=0.0025	*P*=0.7881	*P*=0.8014

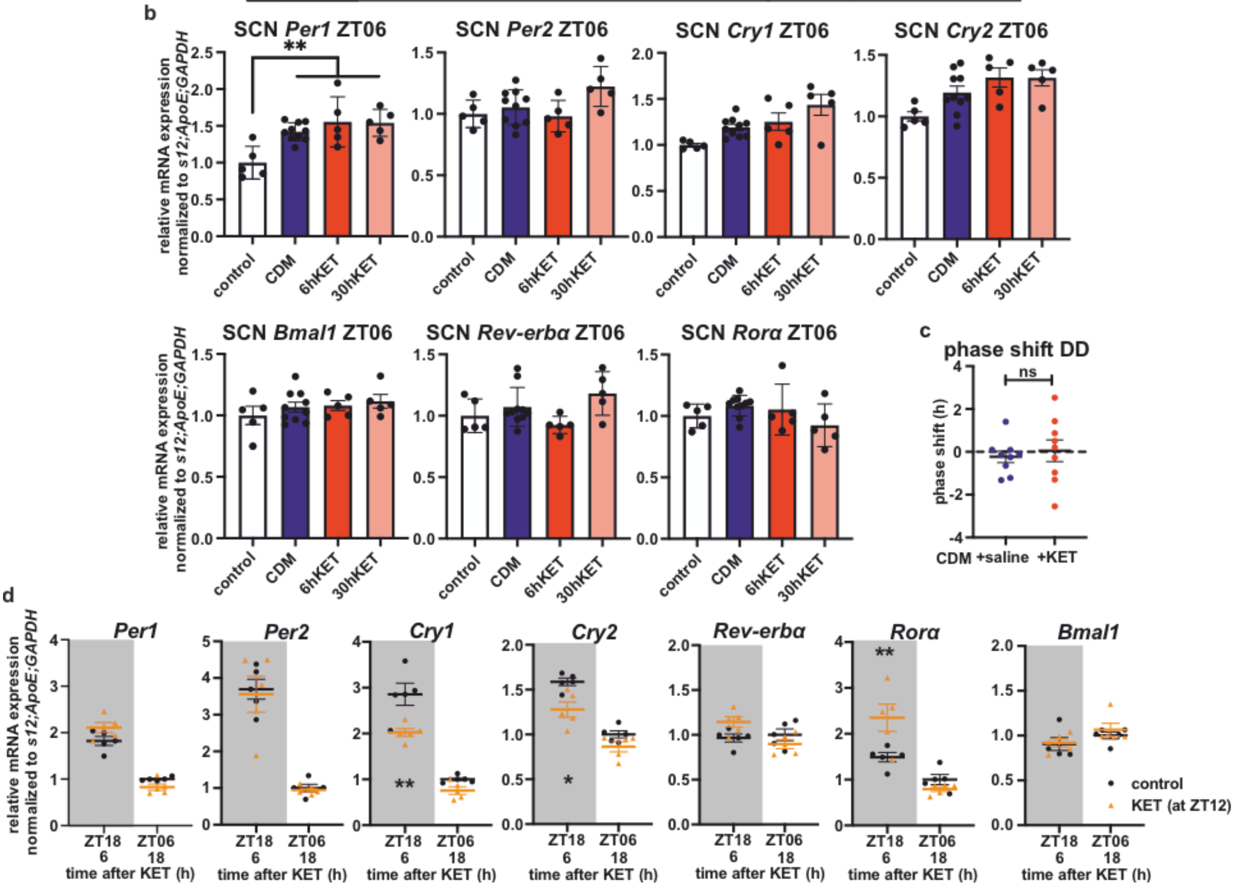

Supplementary Fig. 2

a Comparison of statistical significance for rhythmicity (determined via nonlinear regression sine wave fit and cosinor analyses) and total expression (KET treatment effect, two-way ANOVA); amplitude and acrophase values for naïve (control), CDM and CDM+KET and the statistical significance CDM vs KET and control vs KET (extra sum of squares F test) for each analyzed clock gene in the mPFC. **b** Relative mRNA expression of clock genes in SCN at ZT06 of control (n=5), CDM mice (n=10) and CDM mice 6h (n=5) and 24h (n=5) post KET (injected at ZT00) (one-way ANOVA with Bonferroni post-hoc test: $^{**}P<0.01$). **c** Single saline and/or KET injection at ZT00 have no phase shifting (resetting) effect on the mouse locomotor activity scored in IntelliCage under DD. **d** Relative mRNA expression of clock genes *Per1, Per2, Cry1, Cry2, Bmal1, Rorα, Rev-erbα*, *Rorβ* normalized to *s12*, *ApoE* and *GAPDH* in mPFC samples from naïve mice injected at ZT12 with saline (control) and ketamine (KET) and harvested at ZT18 (6h after injection) and ZT06 (18h post injection) (n=5 mice per group, two-way ANOVA with Bonferroni post-hoc test: $^{*}P<0.05$, $^{**}P<0.01$ CDM vs. KET). Data are presented as mean ±SEM and the individual data points are depicted.

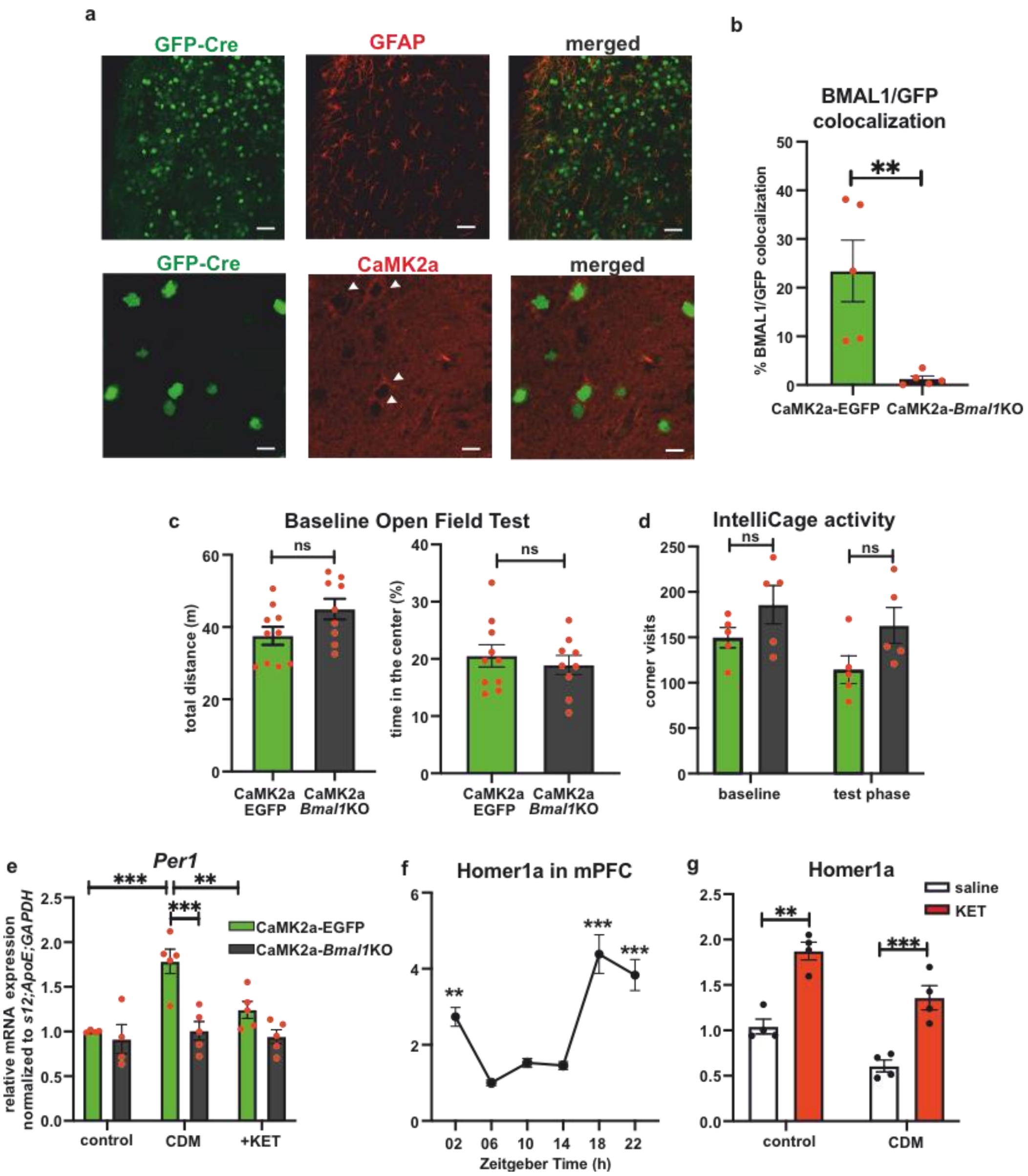

Supplementary Fig. 3

a Representative image of selective viral EGFP-Cre expression selectively in CaMK2a positive neurons of the mPFC (lower panel, green: EGFP-Cre, red: anti-CaMK2a, scale bar, 20 µm). There was no EGFP-Cre expression in GFAP positive astrocytes (upper panel, green: EGFP-Cre, red: anti-GFAP, scale bar, 40 µm). **b** Quantitative data of BMAL1 and EGFP expression colocalization (n=5 per group, two-tailed Student's t-test: ***P*<0.01). **c** Total distance travelled (left) and time spent in the central area (right) in open field test performed at baseline condition of *Bmal1*floxed mice bilaterally injected into the mPFC with control CaMK2a-EGFP or CaMK2a-Cre AAVs (n=10 mice per group, two-tailed Student's t-test). **d** Locomotor and exploratory activity of the mice under 12:12h LD presented as number of corner visits in IntelliCage (n=5 mice per group, repeated measures ANOVA with Bonferroni post-hoc test). **e** Relative mRNA expression of *Per1* in mPFC samples at ZT06 of control (n=4), CDM (n=5) and CDM 24h post KET mice (n=5) (two-way ANOVA with Bonferroni post-hoc test: **P*<0.05, ***P*<0.01, ****P*<0.001). **f** Relative expression of Homer1a in mPFC tissue harvested from naïve (control) mice every 4h at 12:12h LD condition (n=5 mice per group, one-way ANOVA: Bonferroni post-hoc test: ***P*<0.01, ****P*<0.001 control vs. ZT06). **g** Relative expression of Homer1a in mPFC tissue samples at ZT06 in naïve (control), CDM mice, and control and CDM mice 24h post KET (n=4 mice per group, two-way ANOVA with Bonferroni post-hoc test: ***P*<0.01, ****P*<0.001).

Data are presented as mean ±SEM and the individual data points are depicted.

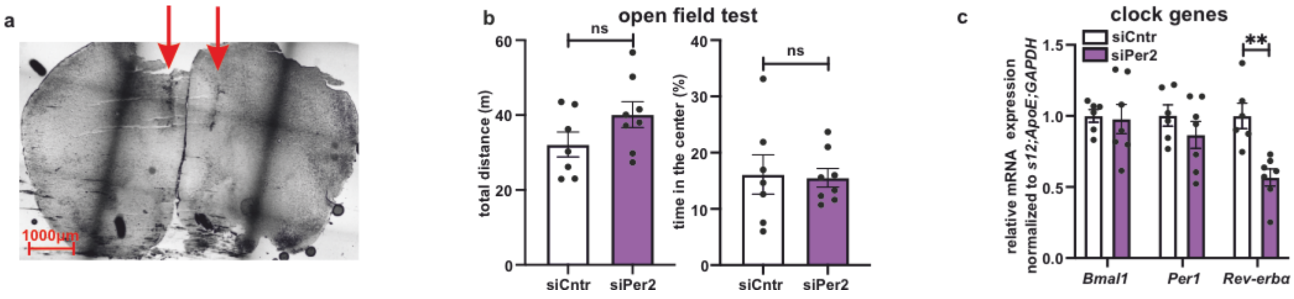

Supplementary Fig. 4

a Representative image showing the siRNA injection site in mPFC. Scale bar, 1000 μm.

b Total distance travelled (left) and time spent in the central area (right) in OFT (n=7 & 8 mice per group, two-tailed Student's t-test).

c Relative mRNA expression of clock genes at ZT06 in mPFC samples of siCntr (n=7 mice) and siPer2 (n=8 mice) injected mice (two-tailed Student's t-test: $**P<0.01$).

Data are presented as mean ±SEM and the individual data points are depicted.

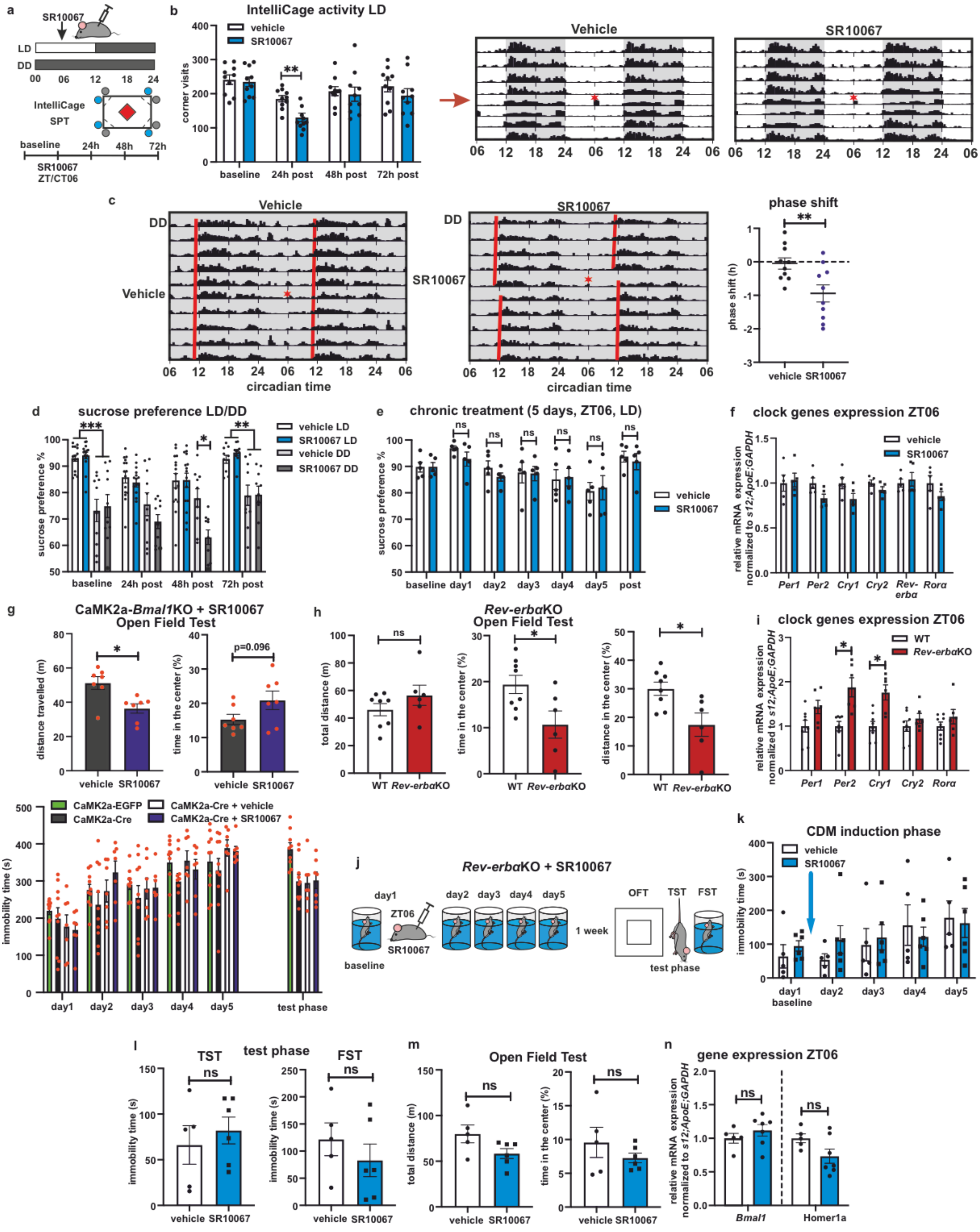

Supplementary Figure 5

Supplementary Fig. 5

a Experimental strategy of the behavioral assessment in the IntelliCage.

b Representative double plotted actogram (right) showing the locomotor activity following vehicle and SR10067 injection at ZT06 in IntelliCage in 12:12 LD condition and locomotor and exploratory activity of the mice (left) under 12:12h LD presented as number of corner visits in IntelliCage at 24h before (baseline), 24h, 48h and 72h post vehicle/SR10067 i.p. injection (at ZT06) (n=8 mice per group, repeated measures two-way ANOVA with Bonferroni post-hoc test: **P<0.01).

c Representative double plotted actogram (left) showing the locomotor activity following vehicle and SR10067 injection at CT06 in IntelliCage in freerunning condition (DD), and phase shift data (right) after vehicle or SR10067 injection (n=10 mice, two-tailed Student's t-test: **P<0.01).

d Sucrose preference assessed in IntelliCage at 24h before (baseline), 24h, 48h and 72h post vehicle/SR10067 i.p. injection (at ZT/CT06) under LD and DD conditions (n=10 mice per group, repeated measures two-way ANOVA with Tukey's post-hoc test: *P<0.05, **P<0.01, ***P<0.001).

e Sucrose preference assessed in IntelliCage after chronic 5 days vehicle/SR10067 i.p. injection (at ZT06) at LD conditions (n=5 mice per group, repeated measures two-way ANOVA with Bonferroni post-hoc test: *P<0.05, **P<0.01, ***P<0.001).

f Relative mRNA expression of clock genes at ZT06 in mPFC samples of vehicle/SR10067 injected WT mice (n=5 mice per group, two-tailed Student's t-test).

g Total distance travelled (left) and time spent in the central area (right) in open field test of vehicle- or SR10067-treated mPFC CaMK2a-BMAL1KO mice (n=7 per group, two-tailed Student's t-test: left, *P<0.05) (top). Immobility time during induction and test phase of CaMK2a-EGFP (n=10), CaMK2a-Cre (n=10) and CaMK2a-Cre mice acutely injected with vehicle/SR10067 (n=7 per group)) on day1 at ZT06 (repeated measures two-way ANOVA with Bonferroni post-hoc test) (bottom).

h Total distance travelled (left), time (middle) and distance (right) spent in the central area in open field test of WT (n=8) and *Rev-erbα*KO (n=6) mice (two-tailed Student's t-test: *P<0.05).

i Relative mRNA expression of clock genes at ZT06 in mPFC samples of WT (n=8) and *Rev-erbα*KO (n=6) mice (two-tailed Student's t-test: *P<0.05).

j Experimental strategy: *Rev-erbα*KO mice underwent the CDM paradigm with vehicle/SR10067 (30mg/kg) injection after first swim session on day 1, with behavioral test phase 1 week later.

k Immobility time during the induction phase of *Rev-erbα*KO mice acutely i.p. injected with vehicle (n=5) or SR10067 (n=6) on day1 at ZT06 (repeated measures two-way ANOVA with Bonferroni post-hoc test).

l Immobility time of TST (left) and FST (right) during the test phase (n=5 & 6, two-tailed Student's t-test).

m Total distance travelled (left) and time spent in the central area (right) in open field test of vehicle- (n=5) or SR10067-treated (n=6) *Rev-erbα*KO mice (two-tailed Student's t-test).

n Relative mRNA expression of *Bmal1* and Homer1a at ZT06 in mPFC samples of *Rev-erbα*KO mice treated with vehicle (n=5) or SR10067(n=6) (two-tailed Student's t-test).

Data are presented as mean ±SEM and the individual data points are depicted.

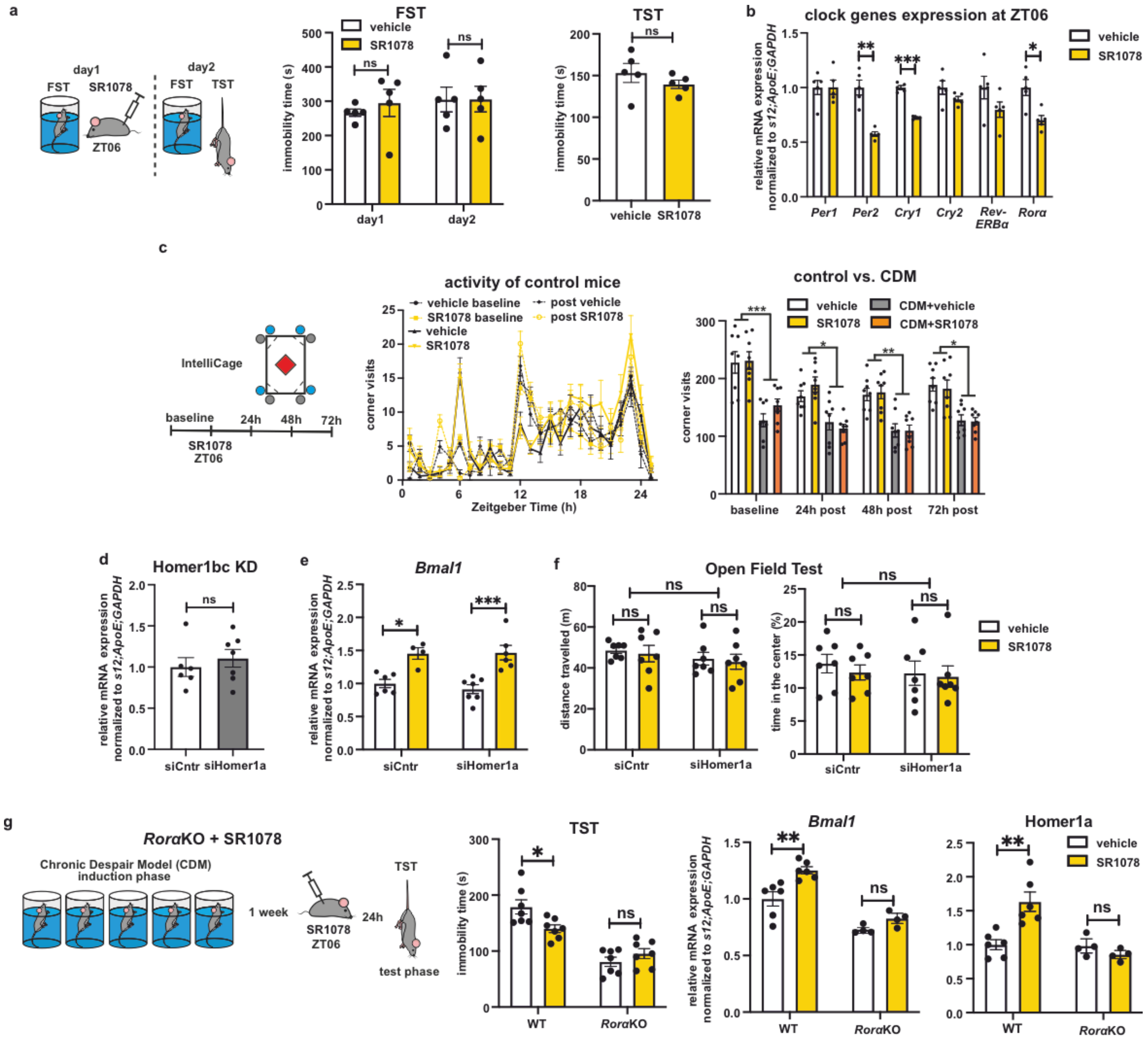

Supplementary Figure 6

Supplementary Fig. 6

a SR1078 has no significant effect on depression-like behavior of naïve mice. Experimental design: WT mice are i.p. injected with vehicle/SR1078 (after the first FST session) and TST and FST tested 24h later. Immobility time in FST (n=5 mice per group, repeated measure ANOVA) and TST (n=5 mice per group, two-tailed Student's t-test: *P*=0.3029).

b Relative mRNA expression of clock genes at ZT06 in PFC samples 24h post vehicle/SR1078 injection (n=5 mice per group, two-tailed Student's t-test: *$P<0.05$, **$P<0.01$, ***$P<0.001$).

c Experimental strategy of the behavioral assessment in the IntelliCage (left). Locomotor and exploratory activity of control (middle left), and control and CDM (right) mice at 12:12h LD presented as number of corner visits in IntelliCage at 24h before (baseline), 24h, 48h and 72h post vehicle/SR1078 i.p. injection (at ZT06) (n=8 mice, repeated measures two-way ANOVA with Bonferroni post-hoc test: *$P<0.05$, **$P<0.01$, ***$P<0.001$). Representative double plotted actogram (centre right) showing the locomotor activity in IntelliCage following vehicle and SR1078 injections at CT06 in freerunning condition (DD).

d Relative mRNA expression of long-form Homer1b/c in mPFC samples after knockdown by siHomer1a or siCntr (n=7 mice, two-tailed Student's t-test).

e Relative mRNA expression of *Bmal1* at ZT06 in mPFC samples 24h post vehicle/SR1078 injection in siCntr (n=6 & 5) and siHomer1a (n=7 & 6) mice (two-way ANOVA with Bonferroni post-hoc test: *$P<0.05$, ***$P<0.001$).

f Total distance travelled (left) and time spent in the central area (right) in open field test of vehicle- or SR1078-treated siCntr and siHomer1a mice (n=7 per group, two-way ANOVA with Bonferroni post-hoc test).

g Experimental strategy (left): *Rorα*KO (n=6) and WT (n=7) mice underwent the CDM paradigm followed by acute i.p. treatment with vehicle/SR1078 (10mg/kg), with behavioral test phase 24h later. Immobility time of TST during the test phase of WT and *Rorα*KO mice after vehicle/SR1078 treatment (middle left, two-way ANOVA with Bonferroni post-hoc test: *$P<0.05$). Relative mRNA expression of *Bmal1* (middle right) and Homer1a (right) in PFC samples 24h post vehicle/SR1078 injection in WT and *Rorα*KO mice (WT n=6 per group, *Rorα*KO n=4 per group, two-way ANOVA with Bonferroni post-hoc test: **$P<0.01$).

Data are presented as mean ±SEM and the individual data points are depicted.

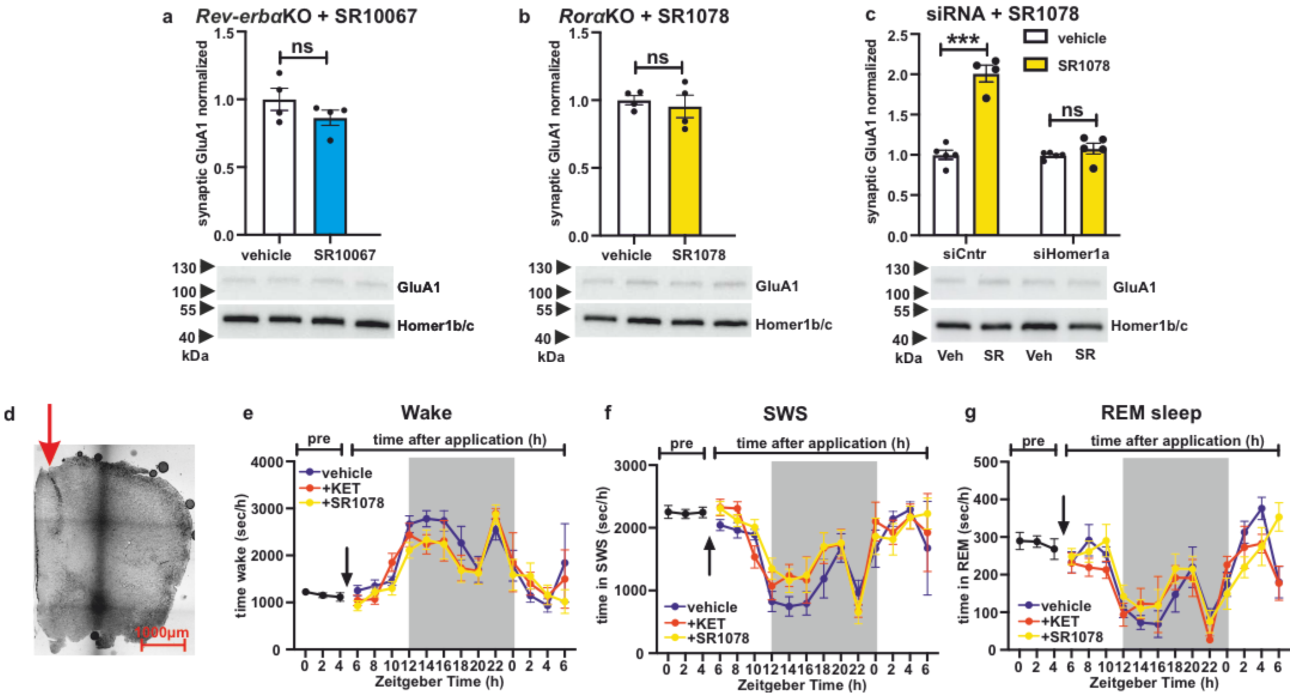

Supplementary Fig. 7

a Quantitative data and representative western blots of synaptic AMPA receptor subunit GluA1 levels in mPFC of *Rev-erbα*KO mice 24h after SR10067 application (n=4 mice per group, two-tailed Student's t-test).

b Quantitative data and representative western blots of synaptic AMPA receptor subunit GluA1 levels in mPFC of *Rorα*KO mice 24h after SR1078 treatment (n=4 mice per group, two-tailed Student's t-test).

c Quantitative data and representative western blots of synaptic AMPA receptor subunit GluA1 levels in mPFC of siCntr and siHomer1a mice 24h after vehicle/SR1078 treatment (n=4 mice per group, two-way ANOVA with Bonferroni post-hoc test, ***P<0.001).

d Representative image showing the LFP electrode insertion site in mPFC. Scale bar, 1000 μm.

e-g time spent in vigilance states wake **(e)**, SWS **(f)** and REM sleep **(g)** per hour, as derived from ECoG/EMG signal, across 12h:12h LD conditions (n=7 mice CDM/vehicle; n=8 mice SR1078; n=7 mice KET).

Data are presented as mean ±SEM and the individual data points are depicted.
