# Supplementary material for: Prefrontal cortex molecular clock modulates development of depression-like phenotype and rapid antidepressant response in mice
Source: Nat Commun. 2024 Aug 23;15:7257. doi: 10.1038/s41467-024-51716-9 (PMC11344080; doi:10.1038/s41467-024-51716-9)
Supplement: Supplementary file 4 — Reporting Summary [file 41467_2024_51716_MOESM4_ESM.pdf]

Corresponding author(s): Tsvetan Serchov

Last updated by author(s): Jul 16, 2024

Reporting Summary

Nature Portfolio wishes to improve the reproducibility of the work that we publish. This form provides structure for consistency and transparency in reporting. For further information on Nature Portfolio policies, see our Editorial Policies and the Editorial Policy Checklist.

Statistics

For all statistical analyses, confirm that the following items are present in the figure legend, table legend, main text, or Methods section.

n/a	Confirmed	
☐	☒	The exact sample size (*n*) for each experimental group/condition, given as a discrete number and unit of measurement
☐	☒	A statement on whether measurements were taken from distinct samples or whether the same sample was measured repeatedly
☐	☒	The statistical test(s) used AND whether they are one- or two-sided<br>*Only common tests should be described solely by name; describe more complex techniques in the Methods section.*
☐	☒	A description of all covariates tested
☐	☒	A description of any assumptions or corrections, such as tests of normality and adjustment for multiple comparisons
☐	☒	A full description of the statistical parameters including central tendency (e.g. means) or other basic estimates (e.g. regression coefficient) AND variation (e.g. standard deviation) or associated estimates of uncertainty (e.g. confidence intervals)
☐	☒	For null hypothesis testing, the test statistic (e.g. *F*, *t*, *r*) with confidence intervals, effect sizes, degrees of freedom and *P* value noted<br>*Give P values as exact values whenever suitable.*
☒	☐	For Bayesian analysis, information on the choice of priors and Markov chain Monte Carlo settings
☒	☐	For hierarchical and complex designs, identification of the appropriate level for tests and full reporting of outcomes
☒	☐	Estimates of effect sizes (e.g. Cohen's *d*, Pearson's *r*), indicating how they were calculated

Our web collection on statistics for biologists contains articles on many of the points above.

Software and code

Policy information about availability of computer code

Data collection	The OFT, TST and FST have been video-recorded with Debut Professional v8.23 video capture software (NCH Software). IntelliCage data were acquired with the PC-based tracking software IntelliCage Plus (TSE Systems). The escape latency in LH was automatically recorded by Packwin 2.0 software (Bioseb). The qRT-PCR data were obtained with the sequence detection software 7300 system SDS v1.3.1 (Applied Biosystems). Western blot images were taken with an Amersham Imager 680 (GE Healthcare). Microscopy images were acquired using ZEN 3.5 software (Carl Zeiss). Electrophysiological data were recorded with Neuvo 64-channel amplifier and ProFusion software package (Compumedics, Australia).
Data analysis	OFT data were analyzed by ANY-maze software (Stoelting Co.). IntelliCage data were analyzed by IntelliCage Plus (TSE Systems). Western blot and immunofluorescent images were quantified with ImageJ 1.53m software (National Institute of Health, USA). Sleep analysis was performed manually using ProFusion software (Compumedics, Australia). ECoG and LFP signals were imported into MATLAB (Mathworks, USA) for spectral analysis using the Chronux (Bokil et al., 2010) and MATLAB Signal Processing (Mathworks, USA) toolkits.

For manuscripts utilizing custom algorithms or software that are central to the research but not yet described in published literature, software must be made available to editors and reviewers. We strongly encourage code deposition in a community repository (e.g. GitHub). See the Nature Portfolio guidelines for submitting code & software for further information.

Data

Policy information about availability of data

All manuscripts must include a data availability statement. This statement should provide the following information, where applicable:

- Accession codes, unique identifiers, or web links for publicly available datasets
- A description of any restrictions on data availability
- For clinical datasets or third party data, please ensure that the statement adheres to our policy

The data sets generated and/or analyzed during the current study can be found in the paper and the supplementary materials. Source data are provided with this paper.

Research involving human participants, their data, or biological material

Policy information about studies with human participants or human data. See also policy information about sex, gender (identity/presentation), and sexual orientation and race, ethnicity and racism.

Reporting on sex and gender	NA
Reporting on race, ethnicity, or other socially relevant groupings	NA
Population characteristics	NA
Recruitment	NA
Ethics oversight	NA

Note that full information on the approval of the study protocol must also be provided in the manuscript.

Field-specific reporting

Please select the one below that is the best fit for your research. If you are not sure, read the appropriate sections before making your selection.

[x] Life sciences [ ] Behavioural & social sciences [ ] Ecological, evolutionary & environmental sciences

For a reference copy of the document with all sections, see nature.com/documents/nr-reporting-summary-flat.pdf

Life sciences study design

All studies must disclose on these points even when the disclosure is negative.

Sample size	The required sample sizes were estimated on the basis of similar past experiences and were verified by power analysis. The number of animals used enabled detection of differences with 80% power and alpha set (Type I error rate) at 0.05. We consult routinely with our biostatistician on site at each step of the project. The exact n is indicated in the figure legends and in Supplementary data table 1.
Data exclusions	Animals were excluded in case of health problems. Mice observed to climb their tails (>10 % of total time) during tail suspension test were excluded from further analysis. For viral or siRNA injection experiments, animals were excluded if the injection did not occur in the correct region or if the expression was not altered. Animals were excluded on the basis of implant problems or poor quality signal, in cases where sleep scoring was unable to be performed due to noise or when spectral analysis confirmed lack of physiological signal. Details are provided in the relevant sections of the Methods, Supplementary Table 1 and Source Data file.
Replication	Most of the behavioral, molecular and electrophysiological results were confirmed by at least one independent replication of the experiments, and results were combined and are presented in the figures. No experimental replications failed to reproduce original results. Supplementary Data Table 1 provides detail on which experiments were replicated, and how many times.
Randomization	For all behavioral, molecular and electrophysiological studies, mice were randomly assigned to groups.
Blinding	The experiments were conducted by completely blinded investigator of the treatment/experimental groups until the data were completely collected.

Reporting for specific materials, systems and methods

We require information from authors about some types of materials, experimental systems and methods used in many studies. Here, indicate whether each material, system or method listed is relevant to your study. If you are not sure if a list item applies to your research, read the appropriate section before selecting a response.

Materials & experimental systems

n/a	Involved in the study	
☐	☒	Antibodies
☒	☐	Eukaryotic cell lines
☒	☐	Palaeontology and archaeology
☐	☒	Animals and other organisms
☒	☐	Clinical data
☒	☐	Dual use research of concern
☒	☐	Plants

Methods

n/a	Involved in the study	
☒	☐	ChIP-seq
☒	☐	Flow cytometry
☒	☐	MRI-based neuroimaging

Antibodies

Antibodies used

Primary antibodies: GluA1 (mouse anti-GluR1-NT, Merck, Cat#MAB2263, RRID:AB_11212678, dilution 1:1000), GluA2 (rabbit anti-GluR2, Merck, Cat#AB1768-I, RRID:AB_2313802, dilution 1:1000), Homer1 (rabbit anti-Homer1, Merck, Cat#ABN37, RRID:AB_11214387, dilution 1:1000), PSD95 (mouse anti-PSD95, Merck, Cat#MABN68, RRID:AB_10807979, dilution 1:1000), PER2 (rabbit anti-mouse Per2, Alpha Diagnostics, Cat#PER21-A, RRID:AB_2161800, dilution1:1000), Histone H2B (mouse anti-Histone H2b, Euromedex, Cat#IG-H2-2A8, dilution 1:500), BMAL1 (rabbit anti-BMAL1, Thermo Fisher Scientific, Cat#PA1-523, RRID: AB_2059614, dilution 1:1000), CaMK2a (mouse anti-CaMK2a, Invitrogen, Cat#Cba-2 13-7300, RRID: AB_86627, dilution 1:1000) GFAP (mouse anti-GFAP, Cy3 Conjugate, Merck, Cat# MAB3402C3, RRID: AB_10916759)

Horseradish peroxidase-conjugated secondary antibodies: sheep anti-mouse, GE Healthcare, Cat#NA931, RRID:AB_772210, dilution 1:20000; donkey anti-rabbit, GE Healthcare, Cat#NA9340, RRID:AB_772191, 1:25000;

Fluorescent secondary antibodies: Alexa Fluor 594 goat anti-mouse, Thermo Fisher Scientific, Cat#A-11032, RRID: AB_2633275, dilution 1:1000; goat Anti-rabbit IgG H&L Cy5 ®, Abcam, Cat#ab6564, RRID: AB_955061, dilution 1:1000

Validation

All primary antibodies were validated by the manufacturer. Information about the use for western blot with murine tissues is provided on the supplier's data sheet and website.

Animals and other research organisms

Policy information about studies involving animals; ARRIVE guidelines recommended for reporting animal research, and Sex and Gender in Research

Laboratory animals

Male and female wild type mice C57BL/6J (Jax Stock#: 000664; RRID: IMSR_JAX:000664) and Bmal1 floxed mutant mice on a C57BL/6J background (B6.129S4(Cg)-Arntltm1Weit/J; Jax Stock#: 007668; RRID: IMSR_JAX:007668), at least 8 week old from Charles River (France and Germany). Male and female Rev-erbαKO mice (B6.Cg-Nr1d1tm1Ven/LazJ; Jax Stock #:018447; RRID:IMSR_JAX:018447) on a C57BL/6J background sourced from the breeding pool maintained at the Chronobiotron, Strasbourg, France, 8-12 weeks old. Male and female RorαKO staggerer mice mice (B6.C3(Cg)-Rorasg/J; Jax Stock #:002651; RRID: IMSR_JAX:002651) on a C57BL/6J background obtained from the breeding pool at Centre de Recherche St-Antoine (CRSA), Sorbonne Université, Paris, France, 8-12 weeks old. The original references are provided in the text.

All mice were at least 8 weeks old at the start of experimental procedures and housed in a temperature- and humidity-controlled environment with ad libitum access to food and water, maintained on a 12h light-dark cycle, unless otherwise stated, where lights on and lights off period mean resting and active phase, respectively, for the mice.

Wild animals

No wild animals were used in the study.

Reporting on sex

Most experiments were performed with both male and female mice. Only females were used for IntelliCage analysis. Supplementary Data Table 1 provides detail on the sex of the animals in each experiment.

Field-collected samples

NA

Ethics oversight

All procedures were performed in accordance with the EU Directive 2010/63/EU for animal experiments and were approved by the animal welfare committee of the Universities of Freiburg (X-20/06R, G-19/44 and G-15/117) and Strasbourg (CREMEAS, APAFIS n° 2020042818477700 and n°36945-2022042219374653v7) as well as by local authorities. Reported in Methods section, under Animals sub-section.

Note that full information on the approval of the study protocol must also be provided in the manuscript.

Plants

Seed stocks	NA
Novel plant genotypes	NA
Authentication	NA
